# Independent replication of a melanoma subtype gene signature and evaluation of its prognostic value and biological correlates in a population cohort

**DOI:** 10.18632/oncotarget.3549

**Published:** 2015-03-12

**Authors:** Jérémie Nsengimana, Jon Laye, Anastasia Filia, Christy Walker, Rosalyn Jewell, Joost J Van den Oord, Pascal Wolter, Poulam Patel, Antje Sucker, Dirk Schadendorf, Göran B Jönsson, D. Timothy Bishop, Julia Newton-Bishop

**Affiliations:** ^1^ Section of Epidemiology and Biostatistics, Leeds Institute of Cancer and Pathology, University of Leeds, Leeds, UK; ^2^ National Heart and Lung Institute, Imperial College, London, UK; ^3^ Yorkshire Regional Genetics Service, Chapel Allerton Hospital, Leeds Teaching Hospitals NHS Trust, Leeds, UK; ^4^ Department of Pathology, University Hospitals Leuven, Leuven, Belgium; ^5^ Department of General Medical Oncology, Leuven Cancer Institute, University Hospitals Leuven, Leuven, Belgium; ^6^ School of Medicine, University of Nottingham, Nottingham, UK; ^7^ Department of Dermatology, Essen University Hospital, Essen, and German Consortium of Translational Cancer Research (DKTK), Heidelberg, Germany; ^8^ Division of Oncology and Pathology, Department of Clinical Sciences, Lund University, Lund, Sweden; ^9^ European Organisation for Research and Treatment of Cancer (EORTC) Melanoma Group, Brussels, Belgium

**Keywords:** molecular subtype, AJCC stage, ROC analysis, vitamin D, telomere length

## Abstract

Development and validation of robust molecular biomarkers has so far been limited in melanoma research. In this paper we used a large population-based cohort to replicate two published gene signatures for melanoma classification. We assessed the signatures prognostic value and explored their biological significance by correlating them with factors known to be associated with survival (vitamin D) or etiological routes (nevi, sun sensitivity and telomere length). Genomewide microarray gene expressions were profiled in 300 archived tumors (224 primaries, 76 secondaries). The two gene signatures classified up to 96% of our samples and showed strong correlation with melanoma specific survival (P=3×10^−4^), Breslow thickness (P=5×10^−10^), ulceration (P=9.x10^−8^) and mitotic rate (P=3×10^−7^), adding prognostic value over AJCC stage (adjusted hazard ratio 1.79, 95%CI 1.13-2.83), as previously reported. Furthermore, molecular subtypes were associated with season-adjusted serum vitamin D at diagnosis (P=0.04) and genetically predicted telomere length (P=0.03). Specifically, molecular high-grade tumors were more frequent in patients with lower vitamin D levels whereas high immune tumors came from patients with predicted shorter telomeres. Our data confirm the utility of molecular biomarkers in melanoma prognostic estimation using tiny archived specimens and shed light on biological mechanisms likely to impact on cancer initiation and progression.

## INTRODUCTION

Considerable efforts have been devoted to improving estimation of cancer prognosis with varying levels of successes. For melanoma, the American Joint Committee on Cancer (AJCC) proposed a staging system based on data from 31,000 melanoma patients [1]. This powerful tool uses histopathological features such as Breslow thickness, presence of microscopic ulceration and mitotic rate that are robust markers of survival, as well as the result of sentinel node biopsy (presence of micrometastases in the sentinel node). Yet, the proportion of the variance in survival explained by the AJCC staging is still suboptimal, with a Receiver Operator Area Under the Curve (AUC) (i.e. proportion of the variance in survival explained by the system) of 74% [2].

Tumor derived gene and microRNA expression profiles have been explored as independent prognostic biomarkers in many cancers, for example breast cancer, colorectal cancer and nasopharyngeal cancer [3-5]. However, melanoma is very heterogeneous [6] and primaries are small, challenging the collection of adequate RNA sampling for inclusion in gene expression studies. A review of published melanoma biomarker reports highlighted a paucity of sufficiently powered and well-designed gene expression studies. However, the few that were compliant with REMARK (REporting recommendations for MARKer prognostic studies [7]) showed the ability of expression signatures to reproducibly predict melanoma prognosis, particularly the most advanced tumors [8, 9]. It was suggested that elucidation of the translational value of these gene signatures requires more research using larger datasets with well-annotated risk factors [8].

Jonsson and colleagues [10] generated a gene signature using 57 stage IV melanomas which they validated in a Swedish cohort of 223 primaries using the cDNA-mediated Annealing, Selection, Ligation and extension (DASL) [11], a technology designed for use with denatured RNA from formalin fixed paraffin embedded (FFPE) tumors [12]. A signature of 503 genes defined 4 molecular classes, which were labeled as “proliferative”, “pigmentation”, “high immune” and “normal-like” reflecting characteristics most notable in terms of gene expression differences between the classes. This signature was subsequently refined to 2 major disease subtypes, the “high” and “low” grade, using a larger signature of 1864 genes [11]. These signatures have not yet been independently replicated in a population cohort.

Molecularly defined high grade tumors were then shown to be thicker, more mitogenic and more frequently ulcerated, all of which are characteristic of poor prognosis. Both the 4-class and the 2-grade categorization defined on the basis of gene expression showed association with prognosis. In multivariable analyses, AJCC stage and molecular grade retained an independent significant prognostic value [11]. There was also an indication that low-grade melanomas were more likely to carry *BRAF* mutations while high-grade tumors were more likely to carry *NRAS* mutations although this subset analysis was based on a small number of tumors [11]. This molecular classification of melanoma appeared therefore to be potentially a valuable tool for understanding the disease biology using FFPE tumor samples and for clinical translation.

The overall aim of the work described here is to assess the relevance of the two and four melanoma subtypes gene signatures developed in a Swedish cohort [11] in a well-annotated, population-based study from the North of England and to seek further evidence that these classes are meaningful by relating them to further patient and tumor characteristics. Specific aims were firstly to replicate these gene signatures in an independent large sample set, and secondly, to assess the added prognostic value.

The description by Jonsson et al. [10, 11] of a 4-class gene signature associated with biological pathways such as proliferation and immune reactions was of note. We therefore also tested the association between this signature and characteristics of the melanoma patients that we have previously reported to be related to melanoma susceptibility pathways, namely telomere length predicted from inherited genetic variation (telomere length score) [13], number of melanocytic nevi [14, 15] and sun sensitivity score [16], to test the hypothesis that different “routes” to melanoma [17] may determine the nature of the tumor. We have also previously reported an association between the 25-hydroxyvitamin D^2^/D^3^ levels at diagnosis (henceforth referred as vitamin D) and outcome [18] and we therefore examined the different molecular tumor sub-types in relation to vitamin D levels at time of recruitment into the Leeds Melanoma Cohort.

## RESULTS

### Quality control

We performed mRNA expression profiling in 357 achieved melanomas using whole genome DASL HT12 v4. This array has 29,354 annotated probes and after examination of those detected by each sample at pvalue<0.05 (median = 14,365, inter-quartile range 12,435 – 15,059), we excluded samples detecting less than 10,000 probes. The final dataset comprised 300 samples: 208 from LMC (204 primaries plus 4 metastases) and 92 from the Chemotherapy study (20 primaries plus 72 metastases). After data normalisation there was high correlation between technical replicates (median 0.97, interquartile range 0.93 – 0.99), notably higher than between non-replicates (median 0.85, interquartile range 0.81-0.88). We aimed to classify these samples using gene signature centroids developed in the Swedish cohort of primary tumors assayed on an earlier version of DASL array (HT8 v3) and that had been filtered during QC to retain 8932 best performing probes [11]. In the present study we have kept all 29,354 probes of the HT 12 v4 array in order to maximize the overlap between probe lists across the two datasets. After merging the probe lists, the overlap was 449/503 (89%) for the 4-class signature and 1584/1864 (85%) for the 2-grade signature. The overlapping probes formed the basis of classification into the 4 category and 2 category schemes.

### Signature replication and association with histology

Demographical and histological data are shown in Supplementary Table S1. All but 4 tumors in the LMC were primaries while 78% were secondaries from the Chemotherapy study. Because of this difference between studies, we present the signature replication in the two datasets separately and combined. Overall, the 4-class signature classified 70 samples as high immune (correlation mean = 0.37, range: 0.12-0.74), 75 as normal-like (correlation mean = 0.43, range: 0.11-0.70), 106 as pigmentation (correlation mean = 0.32, range: 0.10-0.62) and 37 as proliferative (correlation mean = 0.32, range: 0.14-0.62) while 12 (4.0%) were not classifiable (highest correlation to any centroid: 0.03-0.10) (Supplementary Table S2). There was no difference between LMC and Chemotherapy Study in terms of the samples' correlation with the centroids (Supplementary Table S2).

**Figure 1:**
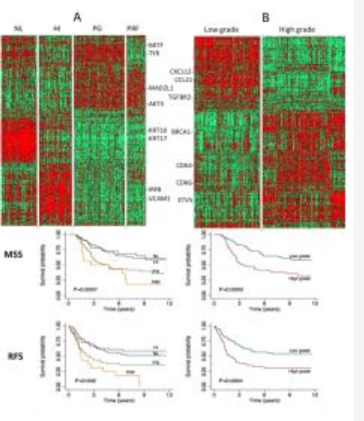
A. Full dataset classification using the 4-class signature and Kaplan-Meir survival curves using primary tumors from LMC (melanoma specific and relapse free survival) HI=high immune, NL=normal-like, PG=pigmentation, PRF=proliferative group. B. Full dataset classification using the 2-grade signature and Kaplan-Meir survival curves using primary tumors from LMC (melanoma specific and relapse free survival). Green = downregulation, red=upregulation.

**Figure 2:**
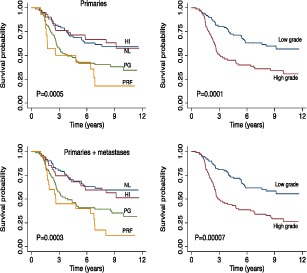
Top panel: Melanoma specific survival of patients with primary tumors from the two datasets Bottom panel: Melanoma specific survival of all patients (primary and metastatic tumors) in the two datasets. Left and right respectively for the 4-class and the 2-grade signatures. HI=high immune, NL=normal-like, PG=pigmentation, PRF=proliferative group.

Figure 1A depicts the molecular classes identified as a heatmap. As reported previously [10, 11], genes such as *MITF* and *TYR* are highly expressed in the pigmentation class, *KRT10* and *KRT17* in the normal-like class, *IRF8* and *VCAM1* in high-immune tumors. Similarly, our data strongly suggested that patients with tumors that exhibit a particular molecular subtype have equally distinct survival profiles (Figure 1A). The patients with tumors classified in normal-like or high immune showed significantly better outcome than those classified as proliferative or pigmentation subtypes. Our data also demonstrated that only 2.7% of tumors classified as normal-like were metastatic compared to 54% of those classified as proliferative (Supplementary Table S3). There was no difference in survival patterns when the analysis was restricted to primary tumors from LMC, to all primaries from both datasets or all primaries and metastatic tumors combined (Figure 2). In addition, our data confirmed that the molecular subtypes are strongly correlated with AJCC stage (P=2.4 × 10^−5^) and 3 of its histological features: Breslow thickness (P= 2.4 × 10^−9^), microscopic ulceration (P=3.0 × 10^−6^) and mitotic rate (P=2.5 × 10^−5^) (Table 1). These results did not change when LMC and chemotherapy data were pooled (Supplementary Table S3).

**Table 1:** Tumor characteristics in sample groups defined by the 4-class and 2-grade signatures in LMC primaries (N=204)

Factor	4-class signature						2-grade signature			
	High immune (N=44)	Normal-like (N=69)	Pigmentation (N=68)	Proliferative (N=14)	Unclassified (N=9)	P ^a^	Low grade (N=88)	High grade (N=78)	Unclassified (N=38)	P ^a^
Sex (Male %)	47.7	42.0	51.5	71.4	55.6	0.23	43.2	51.3	57.9	0.30
Site (limbs %)	58.1	43.5	44.1	21.4	33.3	0.11	47.1	39.7	50.0	0.34
Ulceration (%)	34.3	31.0	70.5	83.3	57.1	3.0×10^−6^	26.1	71.4	55.9	8.9×10^−8^
Vascular invasion (%)	8.3	8.7	19.3	16.7	42.9	0.32	7.9	16.4	25.0	0.14
Tumor infiltrating lymphocytes (%)	36.1	39.1	28.1	41.7	42.9	0.61	44.4	34.2	14.3	0.24
AJCC stage (%)										
I	43.2	43.5	11.8	0.0	11.1	2.4×10^−5^	50.0	6.4	23.7	6.1×10^−9^
II	45.4	49.3	72.1	85.7	88.9		42.0	76.9	68.4	
III/IV	11.4	7.2	16.2	14.3	0.0		8.0	16.7	7.9	
Mutation (%)										
BRAF	47.5	50.0	50.8	25.0	57.1	0.50	53.2	41.9	51.5	0.13
NRAS	20.0	18.3	26.1	25.0	42.9		14.3	27.0	33.3	
Wild type	32.5	31.7	23.1	50.0	0.0		32.5	31.1	15.2	
Age at diagnosis (Median, range)	54.1 (20.0, 72.8)	55.6 (24.6, 74.9)	59.9 (28.7, 78.5)	64.1 (35.1, 76.0)	57.4 (30.1, 76.3)	0.01	55.8 (20.0, 72.8)	59.8 (28.7, 74.8)	56.8 (24.6, 76.3)	0.01
Breslow (mm) (Median, range)	2.0 (1.0, 6.8)	1.9 (0.8, 8.5)	4.0 (1.2, 14.0)	4.2 (2.1, 15.0)	5.3 (1.9, 6.0)	2.4×10^−9^	1.9 (0.8, 7.5)	4.0 (1.2, 15.0)	2.7 (1.3, 12.0)	4.6×10^−10^
Mitotic count/mm^2^ (Median, range)	2.5 (0, 15)	5 (0, 83)	7 (0, 58)	6 (2, 75)	4 (0.5, 18)	2.5×10^−5^	2 (0, 83)	7 (0, 75)	6 (1, 21)	2.5×10^−7^

a Test ignoring the unclassified samples

The 2-grade signature identified 135 samples as high grade (correlation mean = 0.29, range: 0.10 – 0.59), 108 as low grade (correlation mean = 0.34, range: 0.10 – 0.70) and 57 (19.0%) were unclassified (highest correlation range to any centroid: 0.0 – 0.10) (Supplementary Table S2). As expected, the vast majority of normal-like and high-immune tumors were low-grade (90%) while all proliferative and pigment-driven tumors were high grade (100%). Genes such as *CCL21* and *CXCL12* are characteristic of the low-grade while *BRCA1, CDK4* and *CDK6* are markers of the high-grade tumors (Figure 1B). As with the 4-class signature, the high- and low-grade delineation was correlated with AJCC staging (P=6.1x 10^−9^), Breslow thickness (P=4.6x 10^−10^), presence of ulceration (P=8.9 × 10^−8^) and mitotic rate (P= 2.5 × 10^−7^) (Table 1). High-grade tumors were more likely to be metastatic than low-grade tumors (P=7.3 × 10^−4^, supplementary Table S3). Older patients were more likely to have a high-grade disease (P=0.01) while there was no relationship with sex (P=0.3) (Table 1). Patients with high- or low-grade molecular subtypes demonstrated different melanoma specific (MSS) and relapse-free survival (RFS) profiles: this was clearly apparent when the analysis was restricted to primary tumors from LMC (Figure 1B), to all primaries from both datasets or pooled primaries and metastatic tumors (Figure 2).

In melanoma specific survival analysis adjusting for AJCC stage and molecular grade, both predictors showed a strong effect (increasing AJCC HR=2.07, 95%CI 1.40 – 3.06, P=0.0003; molecular grade HR=1.79, 95%CI=1.13 – 2.83, P=0.01). In a multivariable model extended to other risk factors, molecular grade (HR=1.75, 95% CI 1.12 – 2.75, P=0.01), increasing AJCC stage (HR=1.88, 95% CI=1.26-2.83, P=0.002), presence versus absence of vascular invasion (HR=2.44, 95% CI=1.36-4.37, P=0.003) and primary tumor site on the limb *vs.* the rest of the body (HR=0.53, 95% CI=0.34-0.84, P=0.007) retained an independent effect. Supplementary Figures S1–S3 illustrate that when survival analysis is stratified on Breslow thickness AJCC staging, mitotic rate, ulceration, sex, site and age at diagnosis, the molecular grade remains a powerful prognostic tool in each data subset.

### Area under the ROC curves

**Figure 3:**
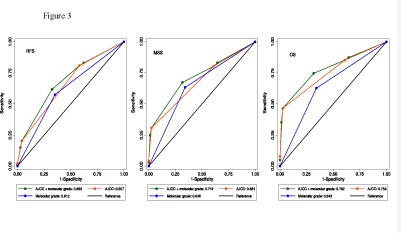
Area under the ROC curves for relapse, death from melanoma and all-causes death

The areas under the ROC curve (AUC) are shown in Table 3 and Figure 3. The AJCC stage has a higher AUC than the molecular grade and when they are combined their AUC reached 0.68 for relapse, 0.72 for death from melanoma and 0.78 for all deaths. The molecular grade increased the AUC by 3%-4% compared to AJCC stage alone and this improvement was significant for all deaths and for death from melanoma (one sided P=0.01 and 0.005 respectively). Inclusion of other risk factors in the model (age, sex, the degree of tumor infiltrating lymphocytes, *BRAF/NRAS* mutation status and tumor regression) did not result in an increase of the AUC. Despite having an independent association with outcome, vascular invasion and tumor site were omitted from ROC analyses due to a large proportion of missing records.

### Association between molecular subtypes and additional phenotypes

In the LMC, the 4-class signature showed an association with germline telomere length score, specifically with the high immune tumors having scores more predictive of shorter telomeres (p=0.03, Table 2). Although not significant there was also a substantial difference in nevus counts between molecular classes, with the high immune group having on average more than twice the number of nevi observed in the proliferative group (50 vs. 22, Table 2). The high/low molecular grade was marginally associated with season-adjusted vitamin D (p=0.04): primary tumors from patients with the lowest level of vitamin D at diagnosis were more likely to be of high grade (Table 2). Although vitamin D was not significantly different between the tumor subtypes defined by the 4-class signature (p=0.15), tumors in the high immune group showed notably the highest levels while the pigmentation group showed the lowest levels.

**Table 2:** Association with additional patients characteristics in the Leeds melanoma cohort primaries (N=204)

Factor	4-class signature						2-grade signature			
	High immune (N=44)	Normal-like (N=69)	Pigmentation (N=68)	Proliferative (N=14)	Unclassified (N=9)	P ^a^	Low grade (N=88)	High grade (N=78)	Unclassified (N=38)	P ^a^
**Telomere length score** **(Median, range)**	−0.15 (−0.25, 0.12)	−0.05 (−0.35, 0.17)	−0.08 (−0.30, 0.17)	−0.03 (−0.21, 0.03)	−0.06 (−0.19, 0.12)	0.03	−0.11 (−0.35, 0.12)	− 0.08 (−0.30, 0.17)	−0.04 (−0.23, 0.17)	0.69
**Mole count** **(Median, range)**	50 (2, 301)	39 (3, 547)	39 (0, 226)	22 (7, 98)	41 (9, 104)	0.12	43 (2, 465)	35 (0, 547)	32 (8, 106)	0.16
**Sun sensitivity score** **(Median, range)**	0.06 (−1.43, 1.23)	−0.11 (−2.00, 1.49)	−0.12 (−1.86, 1.78)	0.19 (−0.76, 0.55)	0.26 (−1.59, 0.88)	0.87	−0.03 (−2.00, 1.49)	−0.07 (1.86, 1.08)	0.14 (−1.56, 1.78)	0.56
**Season adjusted vitamin D** **(Median, range)**	2.0 (−36.1, 39.0)	−4.2 (−41.9, 43.6)	−9.1 (−40.7, 31.4)	−0.8 (−35.4, 29.1)	−6.0 (−26.5, 19.8)	0.15	−4.1 (−39.1, 43.6)	−8.8 (−41.9, 29.2)	−2.6 (−39.9, 31.4)	0.04

a Test ignoring the unclassified samples.

**Table 3:** Area under ROC curves in the full dataset

Factor	Relapse	Deaths from melanoma	All deaths
AJCC stage	0.657	0.681	0.754
Molecular grade	0.612	0.646	0.643
AJCC stage + molecular grade	0.683 ^a^	0.718 ^a^	0.782 ^a^

a Compared to AJCC stage alone, one-sided significance Pvalue=0.06 for relapse, 0.005 for melanoma-caused deaths and 0.01 for all deaths.

## DISCUSSION

The prognosis for stage IV melanoma remains very poor but at last targeted therapies have been shown to have survival benefit [19, 20]. In those treated with the T cell checkpoint inhibitor ipilimumab, approximately 21% of patients survive in the very long term and checkpoint inhibiting PD-1 antibody nivolumab recently demonstrated a 30% increase of 1-year survival rate compared to DTIC in BRAF wild-type advanced melanoma patients [21]. However, Ipilimumab and other immune cell re-activators are associated with considerable toxicity compounded by the fact that patients may require several months of treatment before benefit is seen. There is therefore a great need to identify biomarkers, which predict benefit from such novel therapies. There is currently no adjuvant therapy conferring a survival benefit at acceptable toxicity. However, the rapid progress being made in the treatment of stage IV disease suggests that adjuvant therapies will improve, and better estimates of prognosis and molecular subtypes will also be needed to aid patient selection for such therapies. Current methods of classifying melanoma rely upon histological examination and specific gene mutation testing. It has been recently suggested that the addition of molecular profiling will allow the identification of tumor sub-types possessing both prognostic and predictive significance [22]. The identification of reproducible molecular subtypes is therefore crucial as we move forward into a new therapeutic era.

In this paper we report an independent replication of a published melanoma gene expression signature [11] using data generated from FFPE primary and metastatic melanomas. To our knowledge our data provide a first replication of this signature in a different population. The centroids derived from the Swedish cohort [11] allowed classification of 96% of our samples with the 4-class signature and 81% with the 2-grade signature. High-grade tumors are either more proliferative or driven by pigment-related genes while low-grade tumors are characterised by a high immune activity or a dominance of stromal cells [11]. As in the Swedish cohort, molecular tumor subtypes were not only correlated with patient survival (Figure 1) but also with the main histopathological characteristics of melanoma: AJCC stage, Breslow thickness, mitotic rate, microscopic ulceration and patient age at diagnosis (Table 1 and Supplementary Table S3). Consistent with the Swedish study [11], the molecular grade retained an independent prognostic value in multivariable survival analysis. Our ROC analysis confirmed this added prognostic value with modest but significant increase in the AUC, confirming the well-known high potency of the AJCC system for melanoma (which is notably higher than that of TNM staging of other cancers, see e.g. [2-4]) but also the potential for its improvement.

Historically, it has proved difficult to replicate a number of gene expression signatures due to unreliability of early array technologies, disease heterogeneity, different sample collection methods or bias in the analyses [23]. Despite the known heterogeneity of melanoma [6] and the exclusive use of FFPE tissue, our study shows that advances in transcriptomic assays now allow molecular profiling to be replicated across samples from different populations, in both primary and metastatic tumors. We would propose however, that this replication is only an initial step and that by carrying out similar studies in larger data sets and by incorporating measures such as driver mutation status, gene signatures will be improved. It is the main strength of our sample set that the majority of tumors are population-ascertained (and therefore robust to selection bias) and accompanied by a rich record of phenotypes and genomewide germline genotypes.

It has been suggested that there are different etiological routes to melanoma related to clinical evidence of chronic sun damage (solar keratosis) or large numbers of nevi [17]. We sought to investigate whether particular molecular subtypes were common to patients with characteristics reflecting pathogenic routes to melanoma. Although we noted no associations between the molecular subtypes and sun sensitivity scores derived from hair, eye and skin color, nor with nevus count, we did demonstrate a statistically significant relationship between high immune tumors and a germline-derived polygenic telomere length score. It is intriguing to note that those tumors with a predominantly immune gene expression profile (with better survival) demonstrated shorter telomeres in peripheral blood samples. This finding and published evidence that genetically predicted shorter telomeres decrease melanoma risk [13] suggest a protective role of short telomeres for disease etiology and progression.

It is noteworthy that several studies have demonstrated telomere shortening in subjects with reciprocal reduced long-term immune functioning [24, 25]. Contrasting observations may reflect a fundamental difference in the immunogenicity of melanoma and of the individual tumor sub-types. We hypothesise that in patients whose tumors elicit a long-term immune reaction (thereby resulting in a potentially improved prognosis); we would also expect to see indications of decline in immune response in the form of leukocyte telomere erosion. Contrasting results of telomere length effect on cancer are also common in etiological studies. The increased risk to melanoma and nevi from longer telomeres has been attributed to delayed melanocytes senescence [26], which increases the chance of mutations in the oncogenic *BRAF* gene [27]. Our data must be viewed as preliminary and will be explored in large samples sets. However, the potential to predict telomere length based on molecular stratification of tumors is of clinical importance: longer telomeres were shown to predict the sensitivity of metastatic colorectal cancer to cetuximab, an anti-epidermal growth factor receptor (EGFR) drug [28].

We also tested the relationship between tumor molecular subtypes and season-adjusted vitamin D levels at diagnosis. We found that patients with the lowest levels of vitamin D at recruitment have high-grade tumors and, coincidentally, a worse prognosis (Table 2), supporting the view that vitamin D deficiency might be intrinsically linked to melanoma aggressiveness; although these observations do not demonstrate causality and must be tempered by previous reports that vitamin D levels are higher in fitter, leaner, healthier people and thus the low vitamin D levels noted in a spectrum of medical conditions may be merely acting as a proxy marker of health.

In conclusion, our data provide a first independent validation of previously published melanoma gene signatures and confirm transcriptomic profiling as a valuable biomarker discovery tool for stratified medicine. The associations between disease subtypes with telomere length and vitamin D open up new avenues for understanding biological mechanisms driving tumor development.

## METHODS

### Ethics statement

Investigation was conducted in accordance with the ethical standards and according to the Declaration of Helsinki and according to national and international guidelines and was approved by the authors' institutional review board.

### Study cohorts

Genome-wide gene expression profiles were generated in 357 FFPE tumor blocks from two studies. The first set of 231 were from the Leeds Melanoma Cohort (LMC, ethical approval MREC 1/3/57, PIAG 3-09(d)/2003) comprised of 2184 population ascertained melanoma participants recruited in the North of England since 2000 (median follow up now 8 years) [29-31]. The primary tumors studied from LMC were not selected in that they were the first samples traced and sampled to date. The remaining set of 126 samples was from the Leeds Chemotherapy study (ethical approval 10/H1313/72), which predominantly contains patients with advanced disease from across Europe. The FFPE blocks were sampled horizontally through the most invasive component of the tumor using a 0.6mm Tissue Micro Array (TMA) needle as previously described [29]. Sampling was only performed in those cases where sufficient tumor could be retained within the block after sampling, for future clinical testing.

Detailed histological data were available for both sample sets, derived from the patients' medical records, whilst for those generated from within the LMC more extensive data were available including patient peripheral blood-derived data, such as vitamin D levels measured in a serum sample collected at recruitment using methods described previously [18] and a telomere length score derived from 7 germline Single Nucleotide Polymorphism (SNP) genotypes associated with measured telomere length and previously shown to be associated with risk of melanoma in the general population; increased telomere score implies longer predicted telomere length and increased risk of developing melanoma in the general population [13]. In addition patient questionnaire data were available, including the number of melanocytic nevi 2mm or larger in diameter, a sun sensitivity score derived from factor analysis of hair color, eye color, freckling in childhood, propensity to burn, ability to tan and skin color on the inside upper arm [16].

### Gene expression data production

The mRNA was extracted from the tumor cores following a previously described protocol [29-31] and whole genome gene expression was quantified using the Illumina DASL Human HT12 v4 array. Samples were run in 3 batches and for quality control (QC) purposes, 3 cell lines, 5 control RNAs and 115 technical replicates were added to make a total of 480 arrays across the 3 batches.

### Gene expression data normalisation and quality control

Illumina's GenomeStudio software was used to extract raw data from the image files. In our previous analysis of DASL data the number of transcripts detected per sample was a strong indication of the sample quality [29-31] and accordingly we excluded samples in which a low number of transcripts were detected. Remaining data were background-corrected and quantile-normalised using the package Lumi in R [32]. Normalised full intensity plots and boxplots were examined and the correlations between replicates were compared to non-replicates to assess array performance. Among technical replicates we then excluded the sample with the lowest number of probes detected.

### Replication of the four-class and two-grade gene signature

We used the nearest centroid correlation approach to classify our samples, applying the 4-class and 2-grade centroids from the Swedish cohort of primary tumors (training set) [11]. Each centroid is a vector of average expressions of samples from the training dataset classified into a particular group (high immune, normal-like, pigmentation and proliferative for the 4-class signature; high and low grade for the 2-grade signature). Four-class centroids contain one average value for each of the 503 genes while 2-grade centroids contain one average value for each of the 1864 genes. Each sample was classified separately into (a) one class and (b) one grade by calculating its correlation coefficient with the centroids and allocating the sample to class and grade to which it was the most correlated. A minimum correlation coefficient of 0.10 was required for successful classification, as in the Swedish study. Hierarchical clustering was applied to graphically represent the identified groups using Cluster 3.0 and Java TreeView [33, 34].

### Association between disease subtypes, histopathological variables and other phenotypes

After sample classification into classes and grades we tested the correlation between the defined tumor subtypes, histopathological characteristics, relapse-free survival (RFS) and melanoma-specific survival (MSS). Pearson chi-square and Fisher Exact tests were applied to categorical variables; Mann-Whitney and Kruskall-Wallis tests were applied to continuous variables, Kaplan-Meier plots and log-rank tests were used in univariable survival analysis. The Cox proportional hazard model was applied to multivariable survival analysis adjusting for AJCC stage as a replication of previous reports [11] and with other histological variables. These analyses were conducted in STATA v12 (StataCorp, Texas, USA).

### Area under the Receiver Operator Curves (ROC)

The area under the ROC curves (AUC) was estimated in a logistic regression model predicting relapse, all-cause death and death from melanoma. Univariate AUC was computed for each risk factor and a multivariable AUC was computed in a model that included AJCC stage, molecular grade and traditional risk factors.

## SUPPLEMENTARY MATERIAL, FIGURES AND TABLES


