# Supplementary material for: Independent replication of a melanoma subtype gene signature and evaluation of its prognostic value and biological correlates in a population cohort
Source: Oncotarget. 2015 Mar 12;6(13):11683–93. doi: 10.18632/oncotarget.3549 (PMC4484486; doi:10.18632/oncotarget.3549)
Supplement: Supplementary file 1 [file oncotarget-06-11683-s001.pdf]

Independent replication of a melanoma subtype gene signature and evaluation of its prognostic value and biological correlates in a population cohort

Supplementary Material

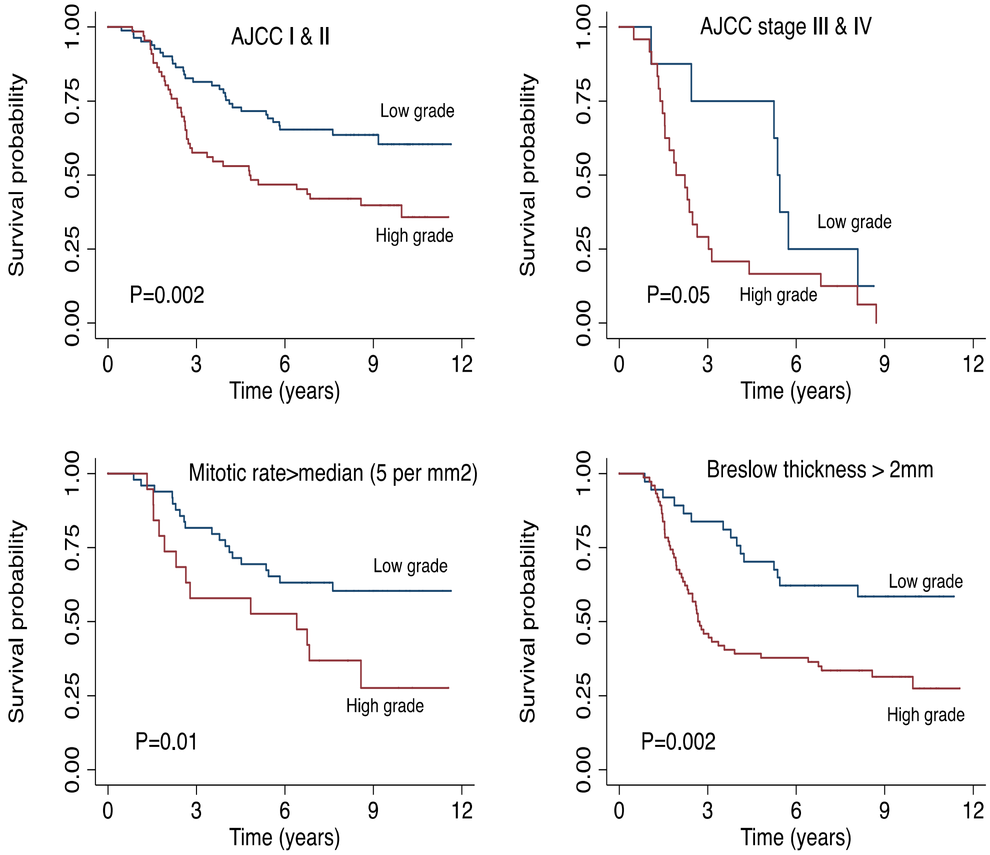

Supplementary Figure S1: Melanoma specific survival of all patients (primary and metastatic tumors) in the two datasets as a function of the 2-grade gene signature stratified on AJCC staging, mitotic rate and Breslow thickness.

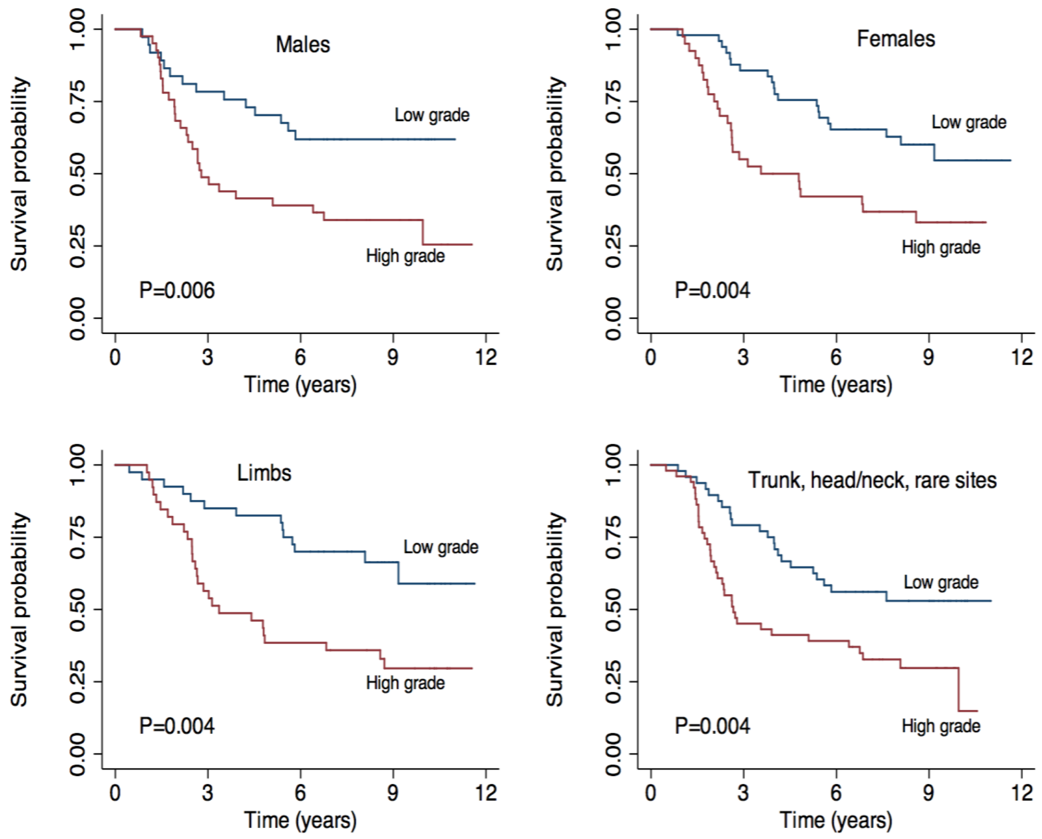

Supplementary Figure S2: Melanoma specific survival of all patients (primary and metastatic tumors) in the two datasets as a function of the 2-grade gene signature stratified on sex and primary site.

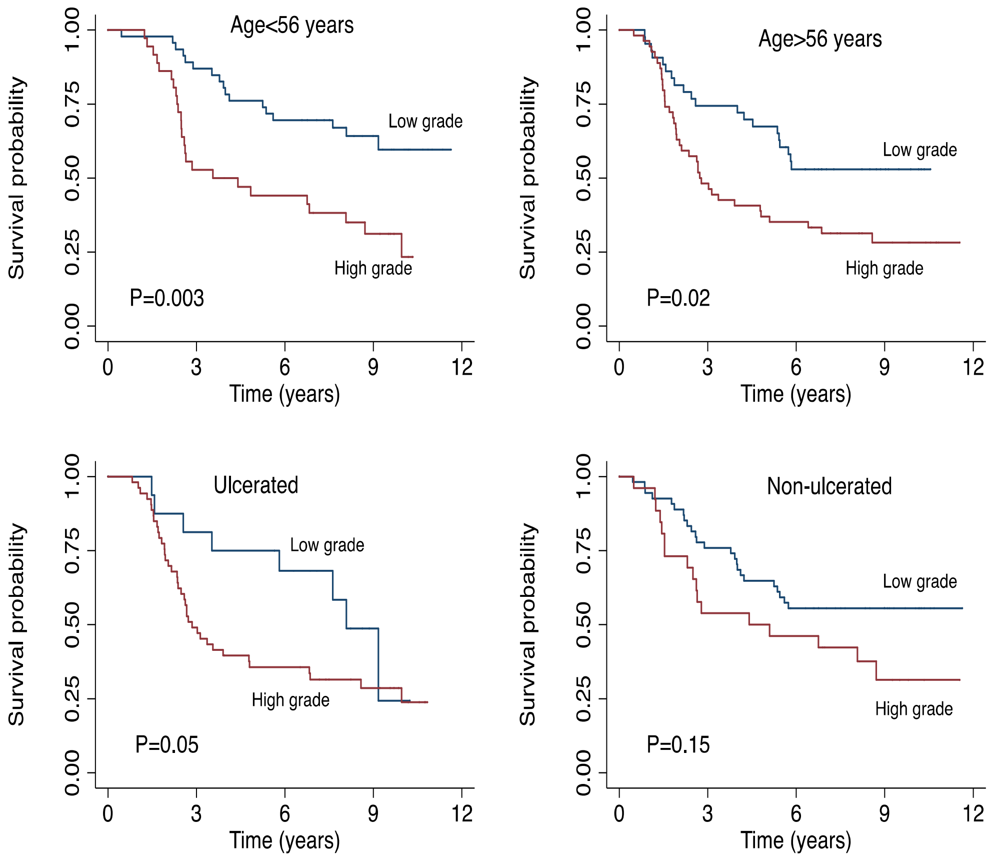

Supplementary Figure S3: Melanoma specific survival of all patients (primary and metastatic tumors) in the two datasets in function of the 2-grade gene signature stratified on age at diagnosis and ulceration.

Supplementary Table S1: Demographical and histological characteristics of the samples from the two datasets

Factor	LMC (N=208)	Chemotherapy study (N=92)
Age, n (%)		
≤50years	65 (31.2)	35 (38.5)
>50 years	143 (68.8)	56 (61.5)
Gender, n (%)		
Female	106 (51.0)	25 (42.4)
Male	102 (49.0)	34 (57.6)
Primary site, n (%)		
Head/neck	31 (15.0)	7 (16.3)
Trunk	68 (32.8)	14 (32.5)
Limbs	93 (44.9)	19 (44.2)
Rare site, sun protected	15 (7.3)	3 (7.0)
Breslow thickness, n (%)		
≤1mm	6 (2.9)	9 (14.3)
1 - 2mm	68 (33.0)	18 (28.5)
2 -4mm	74 (35.9)	19 (30.2)
>4mm	58 (28.2)	17 (27.0)
Ulceration, n (%)		
Present	89 (50.3)	23 (44.2)
Absent	88 (49.7)	29 (55.8)
AJCC stage		
I	58 (27.9)	3 (3.3)
II	123 (59.1)	7 (7.8)
III	25 (12.0)	64 (71.1)
IV	2 (1.0)	16 (17.8)
Tumor types, n (%)		
Primary	204 (98.1)	20 (21.7)
Metastatic	4 (1.9)	72 (78.3)
Mitotic rate, n (%)		-
≤6 mitoses/mm^2	112 (53.8)	
>6 mitoses/mm^2	96 (46.2)	
Mutation status, n (%)		-
BRAF	90 (48.6)	
NRAS	42 (22.7)	
WT	53 (28.7)	
Tumor infiltrating lymphocytes (TILS), n (%)		-
Absent	105 (65.6)	
Present	55 (34.4)	
Vascular/lymphatic invasion, n (%):		-
None	155 (87.1)	
Present	23 (12.9)	
Season-adjusted vitamin D at diagnosis, median (range)	-5.5 (-41.9, 43.6)	-
BMI, n (%)		-
≤30	76 (37.3)	
>30	128 (62.7)	
Sun sensitivity score, median (range)	0.05 (-2.0, 1.8)	-
Mole count, median (range)	39 (0, 547)	-
Telomere length score, median (range)	-0.08 (-0.35, 0.17)	-

Supplementary Table S2: Sample classification: Mean and range of Pearson correlation coefficient between classified samples and the signature centroid of corresponding class or grade from the training dataset (Swedish cohort)

Signature	Classification	Full dataset (N=300)			Leeds Melanoma Cohort (N=208)			Chemotherapy study (N=92)		
		Mean	Range	N	Mean	Range	N	Mean	Range	N
4-class [a]	High immune	0.37	0.12 -0.74	70	0.39	0.12 – 0.74	44	0.34	0.13 0.74	26
	Normal-like	0.43	0.11 -0.70	75	0.43	0.11 -0.70	69	0.39	0.11 – 0.29	6
	Pigmentation	0.32	0.10 – 0.62	106	0.34	0.12 – 0.62	69	0.29	0.10 – 0.42	37
	Proliferation	0.32	0.14 – 0.62	37	0.29	0.14 – 0.60	17	0.35	0.17 – 0.62	20
	Unclassiffied	0.08	0.03 - 0.10	12	0.07	0.03 -0.10	9	0.08	0.08 - 0.09	3
2-grade	Low grade	0.34	0.10 – 0.70	108	0.34	0.10 – 0.70	88	0.32	0.11 – 0.64	20
	High grade	0.29	0.10 – 0.59	135	0.30	0.10 – 0.59	82	0.29	0.10 – 0.46	53
	Unclassified	0.05	0.00 – 0.10	57	0.06	0.00 – 0.10	38	0.04	0.01 – 0.10	19

a: 55 patients (49 from Leeds Meloma Cohort and 6 from Chemotherapy study) were classified in both High immune and Normal-like while 71 patients (41 from LMC and 30 from Chemotherapy study) were classifed in both Pigmentation and Proliferation classes. This large overlap was equally observed in the Swedish cohort and prompted the derivation of the two grade signature with mutually exclusive high and low grades.

Supplementary Table S3: Tumor characteristics in sample groups defined by the four-classes and the two-grade signatures in combined Leeds melanoma cohort and Chemotherapy study

Factor	Molecular class						Molecular grade			
	**High immune (N=70)**	**Normal-like (N=75)**	**Pigmentation (N=106)**	**Proliferative (37)**	**Unclassified (N=12)**	**P [a]**	**Low grade (N=108)**	**High grade (N=135)**	**Unclassified (N=57)**	**P [a]**
Sex (Male %)	57.1	41.9	51.6	57.7	54.6	0.27	46.6	50.0	63.3	0.62
Site (limbs %)	48.9	39.7	34.6	17.6	33.3	0.12	46.8	43.1	44.7	0.60
Ulceration (%)	32.4	32.6	66.2	84.6	57.1	5.8×10^{-6}	27.5	62.1	56.5	3.2×10^{-6}
Vascular invasion (%)	4.3	5.3	10.4	5.4	25.0	0.45	7.5	13.7	22.6	0.29
Tumor type (%)										
Primary	67.1	97.3	73.6	46.0	75.0	2.1×10^{-8}	86.1	67.4	70.2	7.3×10^{-4}
Metastatic	32.9	2.7	26.4	54.0	25.0		13.9	32.6	29.8	
AJCC stage (%)										
I	28.6	41.3	8.6	0.00	8.3	2.6×10^{-11}	42.6	4.5	16.1	2.2×10^{-12}
II	30.0	48.0	50.5	33.3	66.7		36.1	48.5	46.4	
III/IV	41.3	10.7	40.9	66.7	25.0		21.3	47.0	37.5	
Age diagnosis (Median, range)	54.4 (20.0, 85.4)	55.3 (24.6 – 74.9)	56.8 (25.5, 90.7)	61.3 (27.5, 81.4)	57.4 (27.3, 76.3)	0.10	55.6 (20.0, 85.4)	58.7 (25.5, 90.7)	52.2 (24.6, 78.3)	0.06
Breslow (Median, range)	2.0 (0.8, 14)	2.0 (0.8, 8.5)	3.5 (0.3, 15.3)	4.1 (0.8, 15.0)	2.7 (1.9, 6.0)	1.4×10^{-6}	1.9 (0.8, 9.0)	3.6 (0.3, 15.3)	2.6 (0.8, 12.0)	2.6×10^{-7}

a: Test ignoring the unclassified samples.
